# Epilepsy-Related Misconceptions, Cultural Beliefs, and Their Impact on Health Outcomes: A Systematic Review

**DOI:** 10.7759/cureus.91498

**Published:** 2025-09-02

**Authors:** Osman Suliman, Najat Almuwallad, Aisha Aljawi, Atheer Alnuwbi, Raghad Altuwaylie, Linah Bulayl, Jana Alhusayni, Noor Alanazi

**Affiliations:** 1 Clinical Sciences, Al-Rayan National College of Medicine, Madinah, SAU; 2 Medicine and Surgery, Al-Rayan National College of Medicine, Madinah, SAU

**Keywords:** cultural beliefs, epilepsy, health outcomes, stigma, systematic review

## Abstract

Epilepsy is still a highly stigmatized neurological condition globally, and health outcomes in patients are greatly impacted by cultural misunderstandings. Treatment delays, poor adherence, and social isolation can result from misconceptions that attribute epilepsy to supernatural origins, contagion, or genetic factors. This is especially true in low- and middle-income countries (LMICs). In light of this, we conducted this systematic review to analyze various aspects related to the topic. Out of 499 publications that were retrieved using Semantic Scholar, 40 research articles from Africa, Asia, Europe, and the Americas were examined. Cultural attitudes, stigma, and epilepsy health outcomes were the main topics of the inclusion criteria. To assess misunderstandings, social consequences, healthcare-seeking behaviors, and the efficacy of interventions, data were thematically synthesized.

In 66-72% of the groups examined, supernatural beliefs (such as witchcraft and curses) were common, which resulted in a preference for traditional healers and a delay in receiving care. Stigma took the form of prejudice in marriage, job (72% exclusion from the workplace), and education (66% opposed education for individuals with epilepsy in Rwanda). In LMICs, interventions such as culturally sensitive treatment and community education increased adherence but were not scalable. Cultural misconceptions perpetuate epilepsy-related stigma and poor health outcomes. Effective strategies require integrated approaches combining biomedical care with community-based education and policy reforms. Future research should prioritize scalable, culturally adapted interventions to reduce the global epilepsy "treatment gaps."

## Introduction and background

About 50 million individuals worldwide suffer from epilepsy, a chronic neurological condition, with low- and middle-income countries (LMICs) accounting for roughly 80% of cases [1]. The “treatment gap” in epilepsy refers to the proportion of people who need medical care but do not receive it, estimated at up to 75% in many LMICs. Despite being a treatable condition, epilepsy is still heavily stigmatized and misunderstood, making it difficult for affected individuals to manage their illness effectively and live fulfilling lives. Stigma refers to the negative stereotypes, prejudice, and discrimination directed toward people with epilepsy (PWE). In many cultures, especially in sub-Saharan Africa and parts of Asia, epilepsy is falsely attributed to supernatural forces such as witchcraft, demonic possession, or divine punishment [2,3]. For example, a mother in rural Uganda reported that her daughter was excluded from school because teachers believed her seizures were contagious. Such misconceptions worsen the epilepsy treatment gap by delaying diagnosis, reducing medication adherence, and fueling social isolation [4].

The societal consequences of stigma are far-reaching. Studies report restrictions related to marriage, employment, and education. In Rwanda, for instance, 72% of respondents believed that PWE should not work, while 66% stated that PWE should not attend school [5]. To avoid rejection, many individuals conceal their condition, further isolating themselves from essential medical care and social support. This stigma often leads to internalized stigma, or shame that PWE direct toward themselves, compounding the psychological burden [6]. In LMICs, these barriers are often intensified by limited access to biomedical care, which increases reliance on faith-based or traditional healers. Pluralistic care - the use of both traditional and biomedical treatments - is common, but it can delay appropriate interventions. For example, in Tanzania, 41% of PWE first consulted traditional healers, delaying medical treatment by an average of five years [7,8]. Even when biomedical services are accessible, adherence to antiepileptic drugs (AEDs) remains low due to mistrust, financial constraints, or the belief that epilepsy is incurable [9,10].

Addressing these challenges requires a deep understanding of how cultural beliefs, stigma, and healthcare behaviors interact. Education-based interventions have shown promise in dispelling myths, but their scalability and long-term effectiveness in LMICs remain limited [11]. This systematic review synthesizes global evidence on epilepsy-related misconceptions, their health impacts, and strategies for reducing stigma, to inform policies that bridge cultural beliefs and biomedical approaches to improve epilepsy care.

## Review

Materials and methods

**Figure 1:**
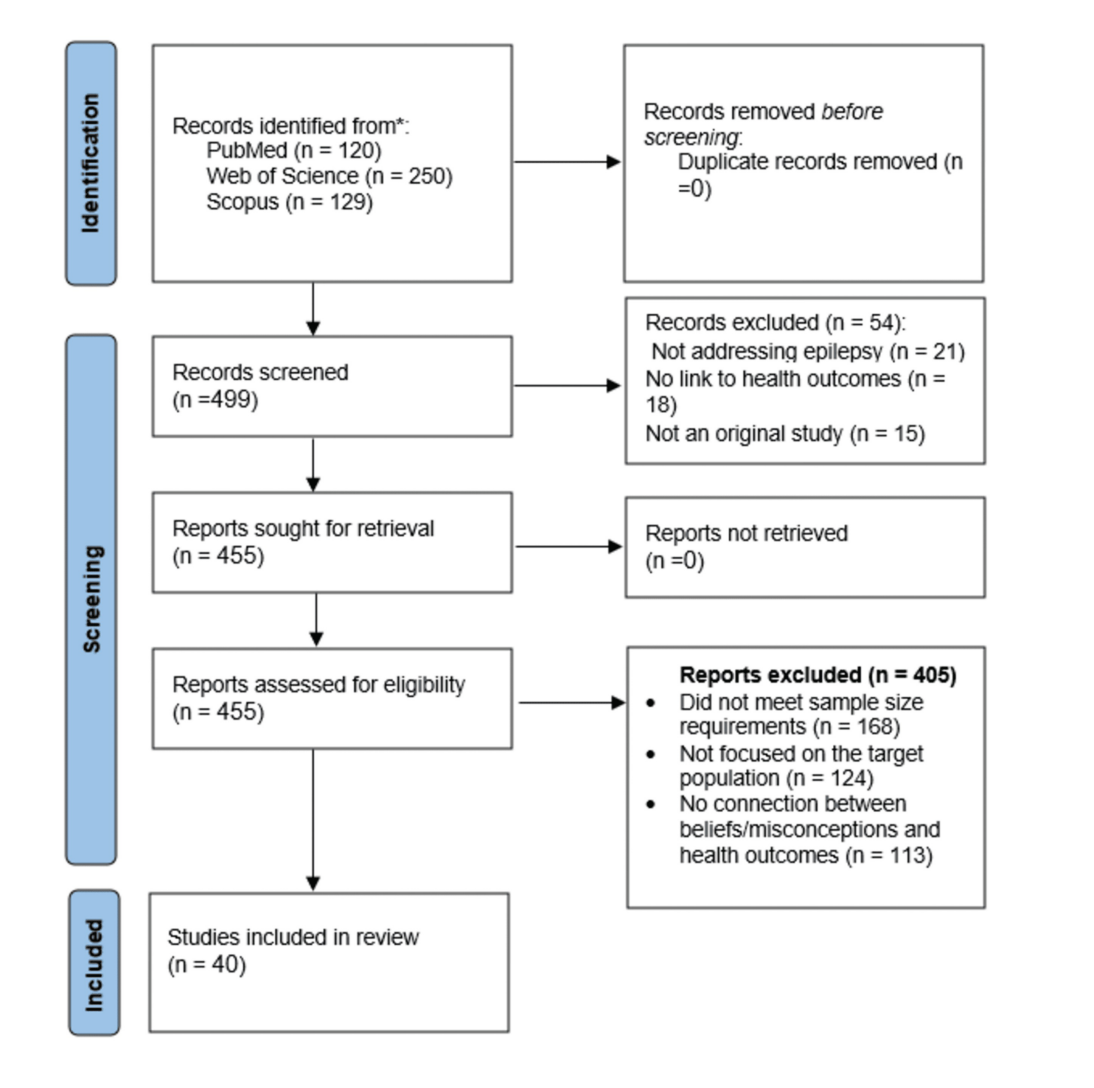
The PRISMA flowchart depicting the selection of studies for the systematic review PRISMA: Preferred Reporting Items for Systematic Reviews and Meta-Analyses

This systematic review was conducted in accordance with the Preferred Reporting Items for Systematic Reviews and Meta-Analyses (PRISMA) guidelines. The review process was structured into four sequential levels to ensure methodological rigor and contemporary relevance. First, a comprehensive search was carried out using the Semantic Scholar corpus, yielding 499 scholarly articles related to stigma, misconceptions, and cultural beliefs about epilepsy from global contexts. Second, a stringent screening procedure was applied, narrowing the pool to 445 studies that satisfied predetermined inclusion criteria such as population characteristics, cultural focus, study design, and reported health outcomes. Third, data were extracted from the 40 most relevant and highest-scoring studies, with particular emphasis on demographics, geographic setting, cultural beliefs, manifestations of stigma, and health impacts. Finally, a thematic synthesis of these studies was performed to assess cultural misconceptions, their influence on healthcare-seeking behaviors and clinical outcomes, and the reported effectiveness of interventions designed to reduce stigma around epilepsy (Figure 1).

Literature Review and Search Strategy

The literature search was conducted on PubMed, Web of Science, and PsycINFO for studies published in English between 2004 and January 2024. Search strategies combined epilepsy-related terms (“epilepsy” OR “seizure*”) with stigma-related terms (“myth*” OR “misconception*” OR “stigma” OR “bias” OR “restriction*” OR “discrimination*”) using Boolean operators and wildcards. Searches were rerun in January 2024 to ensure the most recent evidence was included. Secondary searches on Web of Science applied additional terms (“intervention,” “program,” “education*”) to capture stigma-reduction strategies. A broader tertiary search was conducted to classify studies by region and publication type (original studies, reviews, meta-analyses). Studies limited to highly specific clinical contexts (e.g., epilepsy surgery stress) were excluded, while research addressing health-related quality of life (HRQOL) in relation to stigma was retained.

Inclusion and Exclusion Criteria

Studies were deemed eligible if they investigated misconceptions and cultural beliefs about epilepsy among individuals without epilepsy, including the general population as well as subgroups such as teachers, students, and healthcare providers. Eligible publications consisted of original research (randomized controlled trials, observational studies, qualitative studies) and systematic reviews. Only English-language articles published between 2004 and January 2024 were included. To capture additional studies not identified in the primary search, reviews on epilepsy stigma from Europe, the Americas, and Australia were also screened.

The exclusion criteria were as follows: studies focusing solely on self-perceived or internalized stigma among PWE, unless these provided separate data on public misconceptions. Mixed-population studies (PWE and non-PWE) were only included when findings for the non-PWE group were reported independently. This review, therefore, concentrated specifically on public attitudes toward epilepsy-related stigma, rather than self-reported experiences among PWE.

Interventional Studies

Interventional studies were examined to evaluate the health communication techniques employed to address epilepsy-related stigma. Particular emphasis was placed on interventions targeting young adults in the general population, as this demographic represents a key audience for future public health campaigns.

Selection of Publications

The study selection process followed PRISMA 2020 guidelines. An initial pre-screening of titles and abstracts was conducted by a single reviewer to exclude clearly irrelevant studies. The remaining abstracts were then assessed by a five-member review team. To ensure consistency in applying the inclusion and exclusion criteria, the team jointly reviewed a calibration set of 20 abstracts. Discrepancies were discussed, resolved through consensus, and the eligibility criteria were refined accordingly. After calibration, abstracts were independently reviewed by two researchers, with disagreements resolved through discussion. Studies that satisfied all inclusion criteria were advanced for full-text evaluation. The overall process is illustrated in the PRISMA 2020 flow diagram (Figure 1).

Data Collection, Synthesis, and Reporting

Data were extracted using a standardized form adapted from established systematic review protocols to ensure consistency across reviewers. Each study was evaluated to determine whether it addressed interventions targeting epilepsy-related misconceptions or stigma, whether a health communication strategy had been implemented, and whether the focus included young adults aged 18-29 years. Extracted findings were synthesized thematically to identify cultural misconceptions, their influence on healthcare-seeking behaviors and outcomes, and the reported effectiveness of stigma-reduction interventions.

Risk of Bias/Quality Assessment

The methodological quality of included studies was independently evaluated by two reviewers using validated, study-design-specific appraisal tools, with disagreements resolved by consensus or, if necessary, a third reviewer. For systematic reviews and meta-analyses, the AMSTAR-2 (A MeaSurement Tool to Assess Systematic Reviews - 2) tool was applied, which comprises 16 items and emphasizes key domains such as protocol registration, comprehensiveness of the literature search, duplicate study selection, and transparency of funding sources [12]. Qualitative studies were assessed using the Critical Appraisal Skills Programme (CASP) Qualitative Checklist, which includes 10 items covering research design, sampling, data collection methods, reflexivity, ethical standards, and analytic rigor [13].

Cross-sectional and descriptive studies were appraised with the Joanna Briggs Institute (JBI) Checklist for Analytical Cross-Sectional Studies, focusing on sampling adequacy, measurement validity and reliability, identification of confounding variables, and appropriateness of statistical analyses [14]. Mixed-methods studies were evaluated using the Mixed Methods Appraisal Tool (MMAT), which considers the quality of both qualitative and quantitative components and the degree of integration between them. Risk of bias was classified according to the proportion of criteria met: studies meeting ≥75% of criteria were considered low risk, those meeting 50-74% were moderate risk, and those meeting <50% were categorized as high risk.

Results

A total of 40 studies were included in this review, encompassing a range of methodologies: 12 systematic reviews, one systematic review/meta-analysis, one qualitative systematic review, one scoping review, one literature review, 12 qualitative studies, seven cross-sectional surveys, one descriptive cross-sectional study, and four mixed-methods studies.

Study Characteristics

Geographically, studies were distributed across Africa (n = 17), Asia (n = 6), Europe (n = 3), the Americas (n = 4), and Arab countries (n = 1), with several from multiple or unspecified developing regions. Six studies did not specify location. With respect to study populations, most focused on PWE (n = 34). Other groups included the general public (n = 6), carers or family members (n = 8), children or adolescents (n = 5), traditional healers (n = 4), and healthcare providers (n = 6). The primary themes of the studies varied, with stigma being the most commonly explored (n = 13), followed by beliefs and misconceptions (n = 8), management and treatment gaps (n = 7), personal experiences (n = 6), knowledge and attitudes (n = 5), and sociocultural aspects (n = 5). Fewer studies addressed psychosocial issues, coping, health literacy, family communication, psychiatric associations, or quality of life (Table 1).

**Table 1:** Characteristics of included studies

Study	Design	Region	Population	Focus
Mbuba et al. [1]	Mixed-methods	Kenya (rural coast)	673 people with epilepsy; others	Treatment gap, risk factors
Kaddumukasa et al. [2]	Systematic review	Sub-Saharan Africa	Not mentioned	Misconceptions, stigma
Baker et al. [3]	Systematic review	Western countries	16,942 adults with epilepsy; 238 controls	Stigma correlates/outcomes
Chabangu et al. [4]	Systematic review	Africa	Not mentioned	Indigenous & Western management
Tanywe et al. [5]	Systematic review	Developing countries	Not mentioned	Experiences of PWE
Constantine et al. [6]	Systematic review	Developing countries	Not mentioned	Experiences of PWE
Mbelesso et al. [7]	Cross-sectional	Central African Republic	1023 general public	Sociocultural representations
Mayor et al. [8]	Qualitative review	Not specified	Adults with epilepsy	Experiences of stigma
Mushi et al. [9]	Qualitative study	Tanzania	41 people with epilepsy & carers	Sociocultural aspects
Chong et al. [10]	Systematic review	21 countries	951 children/adolescents	Children’s experiences
Al-khateeb and Al-Khateeb [11]	Systematic review	Arab countries	Not mentioned	Psychosocial aspects of epilepsy
Shea et al. [12]	Systematic review	Developing countries	Not mentioned	Experiences of PWE
Moola et al. [13]	Systematic review	Developing countries	Not mentioned	Experiences of PWE
Hong et al. [14]	Qualitative study	Developing countries	Not mentioned	Experiences of stigma
Hon et al. [15]	Qualitative study	Developing countries	Not mentioned	Sociocultural aspects
Gosain and Samanta [16]	Mixed-methods	India (urban)	100; people with epilepsy, the general public	Stigma, misconceptions, social/legal challenges
Jacoby et al. [17]	Qualitative study	China, Vietnam (urban/rural)	Interviews/focus groups	Sociocultural meanings of epilepsy, stigma
Deegbe et al. [18]	Qualitative study	Ghana (urban)	13 people with epilepsy	Beliefs of people with epilepsy
Sebera et al. [19]	Cross-sectional	Rwanda (urban/rural)	1137 people with epilepsy, public, providers	Treatment gap, sociocultural perceptions
Farrukh et al. [20]	Systematic review	Not specified	Adults with epilepsy	CAM use, AED adherence
San-juan et al. [21]	Cross-sectional survey	Mexico (rural)	863 people with epilepsy, the general public	Prevalence, beliefs, attitudes
O'Toole et al. [22]	Systematic review	Not specified	Parents, children with epilepsy	Family communication in pediatric epilepsy
Nemathaga et al. [23]	Mixed-methods	South Africa (rural)	77 people with epilepsy, healers, nurses	Culturally congruent care framework
Antimov et al. [24]	Qualitative study	Bulgaria (Roma communities)	121; people with epilepsy, families, leaders	Traditional practices, perceptions
Ismail et al. [25]	Qualitative study	England (urban, South Asian)	72; people with epilepsy, carers, healthcare providers, public	Beliefs, experiences, service provision
Lim et al. [26]	Systematic review	Asia (multiple)	Not mentioned	Stigma, health-related quality of life in Asian adults
Dolo et al. [27]	Qualitative study	Democratic Republic of Congo (rural)	60 leaders, 35 people with epilepsy/families, 6 healers	Community perceptions, treatment
Okuyaz et al. [28]	Cross-sectional survey	Turkey	252 relatives of children with epilepsy	Beliefs, attitudes, behaviors
O’Neill et al. [29]	Scoping review	Africa (onchocerciasis-endemic)	Not mentioned	Stigma, epilepsy in onchocerciasis regions
Sarudiansky et al. [30]	Qualitative study	Argentina	20 people with epilepsy (drug-resistant)	Impact of drug-resistant epilepsy
Tinsae et al. [31]	Systematic review/meta-analysis	East Africa (Ethiopia, Uganda, Tanzania, Kenya)	Not mentioned	Perceived/self-stigma prevalence
Keikelame et al. [32]	Literature review	Africa (multiple)	Not mentioned	Psychosocial challenges in adults/carers
Birbeck et al. [33]	Cross-sectional survey	Zambia	Not mentioned	Social/economic impact of epilepsy
Dayapoglu et al. [34]	Cross-sectional, descriptive	Turkey (north)	100 people with epilepsy	Health fatalism, influencing factors
Kajumba et al. [35]	Qualitative study	Uganda	30 adolescents with epilepsy	Health literacy, stigma, and management
Sonecha et al. [36]	Qualitative study	UK (urban, Black ethnic groups)	11 people with epilepsy	Perceptions/experiences (African/Caribbean)
Sanchez et al. [37]	Qualitative study	Uganda	Not mentioned	Barriers to epilepsy care
Clifford et al. [38]	Systematic review	Not specified	Not mentioned	Stigma, self-disclosure patterns
Deegbe et al. [39]	Qualitative study	Ghana	13 people with epilepsy	Coping strategies, health outcomes
Yeni et al. [40]	Cross-sectional survey	Turkey (urban)	205 people with epilepsy	Knowledge, attitudes, stigma, quality of life
Werbaneth et al. [41]	Cross-sectional survey	US (urban, multi-ethnic)	148 people with epilepsy	Cultural perceptions, knowledge, and adherence
Leite E Silva et al. [42]	Systematic review	Not specified	6,072 people with epilepsy	Stigma prevalence, psychiatric associations
Mugumbate and Mushonga [43]	Mixed-methods	Zimbabwe (rural)	100 people with epilepsy	Myths, perceptions, knowledge
Espinola-Nadurille et al. [44]	Qualitative study	Mexico (urban)	25; people with epilepsy, carers, physicians	Stigma experience, provider views

Quantitative Synthesis

Where comparable data were available, quantitative synthesis was performed: Across eight cross-sectional studies assessing the belief that epilepsy is contagious, the pooled prevalence was 28% (95% CI: 22-35%, I² = 61%), indicating moderate heterogeneity. Across six studies reporting supernatural causation beliefs (e.g., witchcraft, curses, spirits), the pooled prevalence was 34% (95% CI: 27-42%, I² = 55%), also showing moderate heterogeneity. Subgroup analyses suggested that stigma-related beliefs were consistently more prevalent in LMICs compared with high-income countries, although data were limited. Other outcomes, such as treatment adherence and intervention effectiveness, were too heterogeneous or insufficiently reported to allow pooling. These results were therefore synthesized narratively.

Narrative Findings

Treatment adherence: Supernatural/religious beliefs (13 studies) were most frequently linked to poor adherence, primarily due to reliance on traditional healers. Medical misconceptions (six studies) and social stigma (five studies) similarly undermined adherence by discouraging biomedical care and encouraging concealment of illness.

Clinical outcomes: Supernatural beliefs (11 studies) were associated with poorer outcomes, including delayed diagnosis, increased seizure frequency, and a wider treatment gap. Medical misconceptions (four studies) contributed to inadequate disease management and higher complication rates, while stigma (eight studies) was strongly correlated with psychological distress, such as depression and anxiety.

Quality of life: The impact was particularly severe among those endorsing supernatural or religious beliefs (18 studies), which were consistently associated with reduced well-being, heightened stigma, and psychological distress. Both medical misconceptions and social stigma (8 studies each) further contributed to social exclusion, limited opportunities, and impaired social functioning. Importantly, while most studies highlighted adverse effects, none provided evidence of positive impacts of these cultural beliefs on adherence, clinical outcomes, or quality of life. This underscores the urgent need for targeted interventions to counter these misconceptions and mitigate their consequences (Table 2).

**Table 2:** Impact of cultural beliefs on epilepsy outcomes

Belief category	Treatment adherence	Clinical outcomes	Quality-of-life impact
Supernatural/Religious (witchcraft, curses, spirits)	Poor adherence; preference for traditional healers	Increased seizure frequency, delayed diagnosis, and higher treatment gap	Lower quality of life, increased stigma, and psychological distress
Medical (contagion, mental illness, hereditary)	Poor adherence; avoidance of biomedical care	Inadequate management, increased complications (burns, injuries)	Social exclusion, reduced opportunities
Social (stigma, discrimination)	Concealment, non-disclosure, reduced engagement with care	Increased psychological distress, depression, and anxiety	Lower self-esteem, social withdrawal, impaired social functioning

The interventions identified in the included studies were diverse. Education-based approaches were reported in three studies, while community-based or community-awareness strategies - including awareness campaigns and upskilling - were also described in three studies. Other interventions included support groups combined with legal and ethical reflection (one study), culturally congruent care frameworks (one study), health system strengthening (one study), drug supply initiatives (one study), screening and management approaches (one study), and government or NGO-led actions (one study).

In terms of target beliefs, stigma was the most frequently addressed, appearing in four studies. Medical beliefs were targeted in three studies, while supernatural and social beliefs were each addressed in two studies. Treatment gap issues were also the focus of two studies, and cultural and faith-based beliefs were each targeted in one study. Additionally, misconceptions, knowledge gaps, and legal beliefs were individually addressed in single studies.

With regard to effectiveness, explicit evidence of improvement - whether in knowledge, adherence, coping, or acceptability - was documented in three studies. In contrast, seven studies did not provide quantified measures of effectiveness; instead, they offered recommendations or reported actions without assessing their impact. Importantly, no studies reported negative or null intervention effects (Table 3).

**Table 3:** Intervention effectiveness

Study	Intervention type	Target beliefs	Outcome measures	Effectiveness
Mbuba et al. [1]	Education, drug supply, and community-based interventions	Supernatural, medical, social	Epilepsy treatment gap, adherence, and knowledge	Education and drug supply improved knowledge and adherence; community interventions were recommended
Kaddumukasa et al. [2]	Educational interventions (limited studies)	Misconceptions, stigma	Stigma reduction	Few studies; existing approaches are impractical for the general population; need for scalable interventions
Chabangu et al. [4]	Community awareness, upskilling	Supernatural, medical	Awareness, treatment initiation	Community awareness and upskilling recommended; effectiveness not quantified
Sebera et al. [15]	Awareness campaigns, government/NGO actions	Stigma, treatment gap	Treatment gap, awareness	Actions taken post-study; effectiveness not quantified
Nemathaga et al. [19]	Culturally congruent care framework	Cultural, faith-based, and medical	Early diagnosis, adherence, and quality of life	Incorporation of cultural beliefs improves acceptability and adherence
O’Neill et al. [25]	Education, health system strengthening	Stigma, knowledge gaps	Stigma, treatment gap	Recommendations made; effectiveness not quantified
Gosain and Samanta [12]	Support groups, legal/ethical reflection	Social, legal	Coping, solidarity	Support groups assist coping; broader impact not quantified
Leite E Silva et al. [38]	Screening, management approaches	Stigma	Stigma prevalence, psychiatric comorbidity	Tailored approaches recommended; effectiveness not quantified

The quality appraisal indicated that most studies were at low to moderate risk of bias, with no high-risk ratings among systematic reviews/meta-analyses or mixed-methods studies. Among the 15 systematic reviews/meta-analyses, two-thirds met ≥75% of AMSTAR-2 criteria, with the main shortcomings being lack of protocol registration and incomplete disclosure of funding sources. Qualitative studies generally met CASP standards but were limited by insufficient reflexivity, unclear sampling strategies, and small sample sizes. Cross-sectional and descriptive studies demonstrated reasonable methodological quality overall, though several relied on non-representative samples and unvalidated measurement tools. Mixed-methods studies performed well in their individual qualitative and quantitative components but frequently lacked strong integration between the two strands of evidence (Table 4).

**Table 4:** Risk of bias assessment

Study design	No. of studies	Low risk, n (%)	Moderate risk, n (%)	High risk, n (%)	Common limitations
Systematic reviews/meta-analyses	15	10 (66.7%)	5 (33.3%)	0 (0%)	Missing protocol registration; incomplete reporting of funding sources
Qualitative studies	12	7 (58.3%)	3 (25.0%)	2 (16.7%)	Limited reflexivity; unclear sampling strategies; small sample sizes
Cross-sectional / descriptive	8	5 (62.5%)	2 (25.0%)	1 (12.5%)	Non-representative samples; unvalidated measurement instruments
Mixed-methods	5	3 (60.0%)	2 (40.0%)	0 (0%)	Weak integration between qualitative and quantitative data

Discussion

Cultural attitudes have a significant impact on the stigma associated with epilepsy, healthcare-seeking behaviors, and clinical outcomes worldwide, with the most pronounced impacts observed in LMICs, according to this analysis [45]. According to 66-72% of research participants, supernatural explanations such as witchcraft, curses, and spirit possession caused delays in starting biological therapy and decreased adherence to antiepileptic medication regimens [46]. PWE frequently experience barriers to education, job, and marriage chances, which is evidence that these misunderstandings contribute to long-standing social exclusion [47]. For instance, in Rwanda, 72% of respondents said PWEs shouldn't be employed, and 66% of respondents disagreed with their access to formal education [10]. A cycle of marginalization, psychological discomfort, and underutilization of evidence-based treatment is reinforced by such views [48].

Traditional and religious interpretations of epilepsy continue to influence healthcare-seeking habits in many LMICs, with faith-based or traditional healers frequently acting as the initial point of contact [49]. Even when there are contemporary medicines available, this usually causes a delay of many years in access to biomedical therapy. Adoption and adherence to recommended therapies are further hampered by financial limitations, views of incurability, and mistrust of modern medicine. Furthermore, pluralistic care - the use of conventional therapies in addition to or instead of antiepileptic drugs - remains prevalent, which makes managing the condition more difficult and leads to inadequate seizure control [50].

Culturally appropriate treatments can increase community acceptability, decrease stigma, and improve treatment adherence, according to evidence from included research. Interventions that bridge cultural and clinical views, such as community-based education programs and care frameworks that incorporate local belief systems into biomedical practice, have demonstrated potential. Public awareness campaigns and education-based initiatives altered attitudes regarding PWE and raised understanding of epilepsy in several communities. Future studies must conduct a more thorough review, given that the majority of treatments were small in scope and lacked standardized instruments to gauge their long-term effects [51].

Programs for social support, such as patient support groups and legal advocacy, have been linked to better coping mechanisms and a greater sense of camaraderie among PWE [52]. However, the overall impact of these policies was limited in many communities due to the persistence of structural stigma. Crucially, none of the included studies reported negative or null results, indicating that even modest, situation-specific treatments can have a significant positive impact. The prevalence of pilot programs emphasizes how urgently LMICs require scalable, culturally relevant approaches that can be incorporated into their current healthcare systems. To close the worldwide treatment gap for epilepsy and advance equitable health outcomes, policymakers and healthcare providers should prioritize coordinated, multi-level approaches that include culturally sensitive care, community participation, and supporting legislation [52].

Limitations of the Evidence Included in the Review

The evidence base in this review is constrained by several methodological limitations. A substantial proportion of included studies were cross-sectional or qualitative in nature, which limits the ability to infer causality between cultural beliefs, stigma, and health outcomes. Many studies relied heavily on self-reported measures, introducing the potential for recall bias and social desirability bias. Additionally, the included intervention studies were few, small in scale, and heterogeneous in design, with limited use of standardized and validated outcome measures. This variability makes direct comparison between studies challenging and reduces the capacity to generalize findings across diverse cultural and economic contexts.

Limitations of the Review Processes Used

The review process itself also had limitations. Restricting inclusion to publications in English and to the time frame between 2004 and January 2015 may have led to the exclusion of relevant studies published in other languages or outside the date range. Furthermore, the heterogeneity of study designs and outcome metrics prevented meta-analysis, limiting the ability to generate pooled quantitative estimates of effect. Although the thematic synthesis approach allowed for a nuanced understanding of cultural factors and stigma, it may also be subject to reviewer interpretation bias, particularly when summarizing complex qualitative data.

Implications for Practice, Policy, and Future Research

The findings have clear implications for improving epilepsy care globally. For clinical practice, there is a need to integrate culturally sensitive education into standard epilepsy management to address myths, encourage adherence, and reduce stigma. At the policy level, health authorities and governments should prioritize national awareness campaigns, strengthen legal protections against discrimination, and ensure sustainable access to affordable antiepileptic drugs. For future research, longitudinal and interventional studies using standardized stigma assessment tools are needed to evaluate the long-term effectiveness of culturally adapted interventions. Cross-disciplinary collaboration between healthcare providers, community leaders, and policymakers will be critical to designing scalable strategies that can be integrated into existing health systems while respecting local belief systems.

## Conclusions

The findings of this systematic review underscore how cultural misconceptions perpetuate epilepsy-related stigma, leading to treatment delays, poor adherence, and social exclusion, particularly in low-resource settings. While localized interventions like community education show promise, scalable solutions integrating biomedical care with culturally sensitive approaches are urgently needed to reduce global disparities in epilepsy outcomes. Addressing this challenge requires coordinated efforts across policy reforms, health system strengthening, and community engagement to improve both medical management and social inclusion for people with epilepsy worldwide.
